# Development of an Educational Program for Non-Professional Soccer Coaches in Charge of Community-Based Soccer in Men with Prostate Cancer: a Qualitative Study

**DOI:** 10.1186/s40798-018-0147-y

**Published:** 2018-07-13

**Authors:** Eik Dybboe Bjerre, Mette Leth, Nanna Maria Hammer, Julie Midtgaard

**Affiliations:** 1 The University Hospitals’ Centre for Health Research, Copenhagen University Hospital Rigshospitalet, Department 9701, Blegdamsvej 9, DK-2100 Copenhagen Ø, Denmark; 2 Department of Public Health, University of Copenhagen, Øster Farimagsgade 5, P.O. Box 2099, DK-1014 Copenhagen K, Denmark

**Keywords:** Football, Cancer survivor, Community, Exercise, Rehabilitation

## Abstract

**Background:**

While clinical trials have demonstrated the benefits of structured exercise for prostate cancer survivors, few attempts have been made to investigate and implement sustainable community-based exercise programs supporting adoption of long-term physical activity behavior. Against this background, the aims of this study was to explore the perspectives of experts and stakeholders on the development of a training course and intervention manual used to support the delivery of community-based soccer training in men with prostate cancer (the FC Prostate Community [FCPC] trial).

**Methods:**

A two-step qualitative design including triangulation of methods, data sources, and researchers. Step 1 comprised key informant interviews with clinical and scientific experts (*n* = 4). Step 2 included stakeholder focus group interviews with nurses (*n* = 5), non-professional soccer coaches and club representatives (*n* = 5), and prostate cancer survivors (*n* = 7).

**Results:**

Four themes emerged from the analysis of the key informant interviews: *The Coach’s Qualifications*, *Structure of the Training*, *Prevention of Injuries*, and *A Non-Patient Environment*, which informed development of the training course and intervention manual. The stakeholders added the importance of clarifying the *Responsibility of the Coach*, the value of *Positive Competition*, and *Social Inclusion* of the prostate cancer survivors in the club. Based on these results, we present the final templates for the training course and intervention manual.

**Conclusions:**

No general set of rules or safety measures to promote or optimize the delivery of community-based exercise in cancer survivors is recommended. However, the general principles related to the necessary clarification of the coach’s responsibility in relation to the prevention and management of injuries and participant adherence through a non-patient environment may be transferable to the training and education of other groups of lay persons in charge of delivering exercise interventions to other clinical subpopulations in a non-hospital setting.

## Key points

- Hospital-based exercise training programs supervised by health professionals have limited potential for physical activity developing into a permanent lifestyle in clinical populations.
- Key experts and stakeholders identified knowledge about prostate cancer, empathy, and ability to generate team spirit as important qualifications in instructors/coaches in charge of community-based soccer to men with prostate cancer.
- Lay coaches delivering community-based soccer to men with prostate cancer are expected and requested to function as an authority and provide for the men’s safety while simultaneously serving as a soccer coach in the traditional sense and capitalizing on the setting outside the hospital.

## Background

Early detection and advances in treatment have resulted in substantial improvements in prostate cancer survival rates. However, as a result of often prolonged hormone suppression, prostate cancer survivors are at high risk of treatment-related late effects that can impair bone health and loss of muscle mass [1], redisposing to serious health conditions and poor quality of life exacerbated by sedentary behavior. In Sweden and the USA, men diagnosed with prostate cancer are less likely to die from prostate cancer than from another cause, e.g., ischemic heart disease and cerebrovascular disease [2]. Because many of these other causes of death are preventable through changes in lifestyle, development of novel interventions that target lifestyle factors are considered imperative [3]. Several clinical trials [4–8] indicate that strength training or a combination of aerobic and strength training has a positive effect on body strength, fatigue, and quality of life in prostate cancer survivors. To date, however, most studies investigated highly structured and professionally supervised on-site exercise training programs. While promising, these studies have limited potential for physical activity developing into a new and preferred permanent lifestyle essential for the sustainment of the effects long term, including a potentially protective effect on all cause- and prostate cancer-specific mortality [9].

Burgeoning evidence suggests that recreational soccer, i.e., non-tournament-based small-sided games, may constitute a novel intervention for patients living with chronic diseases. We previously examined the safety and efficacy of 12 weeks of recreational soccer in men with prostate cancer undergoing androgen deprivation treatment (The FC Prostate RCT) [10]. The results published to date show that professionally supervised soccer training may improve lean body mass, muscle strength, and bone health [11, 12]. Moreover, we documented that soccer is regarded by participants as a welcome opportunity to regain control and take responsibility for their own health in a male-oriented setting without assuming the patient role [13]. However, because the intervention was carried out and evaluated in a hospital-based setting characterized by substantial professional resources and a highly selected group of patients, the external validity of previous findings are limited, and the effects of the intervention when delivered in a real-life setting, i.e., in local soccer clubs, remain uncertain. To meet the challenge of managing the long-term health consequences of prostate cancer treatment, including sustainable provision of novel survivorship care strategies, we initiated the FC Prostate Community (FCPC) trial to examine the effectiveness of community-based soccer training in men diagnosed with prostate cancer [14]. By making use of existing structures for physical activity, i.e., local soccer clubs, the trial responds to the challenges related to limited accessibility to facilities, resources, and equipment, pivotal to cancer survivors’ long-term maintenance of physical activity [15]. However, given its base in the community, the FCPC intervention implies recruitment of non-professional soccer coaches with no prior clinical experience and training. Against this background, *the purpose of this study* was to explore the perspectives of experts and stakeholders on the development of a curriculum for educating non-professionals in the delivery of community-based soccer in men with prostate cancer.

## Methods

### Design

The study was designed as a two-step, theoretically flexible qualitative study that included triangulation of methods, data sources, and researchers (i.e., one male [EDB] and three females [ML, NMH, and JM] representing three different disciplines: physiotherapy [EDB], occupational therapy [ML and NMH], and psychology [JM]). The purpose of step 1, which included four key informant interviews (*n* = 4) with clinical specialists and scientific experts, was to develop an outline for the curriculum, which would be validated and further developed in step 2, which comprised three focus group interviews with various stakeholders (i.e., groups involved in and affected by the study and ultimate implementation of the intervention).

### Sampling

The participants were purposefully chosen to secure recruitment of information-rich cases [16], i.e., informants with specific knowledge, experience, or interest in relation to the study aims. Participants in step 1 were thus recruited among experts with extensive knowledge of soccer, prostate cancer, and male psychology, respectively, whereas the purposeful selection of participants in step 2 involved recruitment of representatives from the three main stakeholder groups in the FCPC trial. Tables 1 and 2 present the specific inclusion criteria, recruitment procedure, and characteristics of informants included in steps 1 and 2.

**Table 1:** Area of expertise, recruitment, and characteristics of key informants in step 1

	Informant	Area of expertise	Recruitment	Interview setting	Informant characteristics
STEP 1 EXPERTS (*N* = 4) (key informant interviews)	Professor Peter Krustrup (PK)	Extensive knowledge and experience in human physiology and soccer research	Personal contact at a meeting	The informant’s office	Professor of Team Sport and Health; main research areas include muscle physiology, prevention and treatment of lifestyle diseases through physical activity, and training and testing in elite sport.
	Professor Klaus Brasso (KB)	Extensive experience with clinical research and medical treatment of prostate cancer patients	Personal email	Meeting room, Department of Urology, Copenhagen University Hospital, Rigshospitalet	Professor, senior consultant/resident, M.D., Ph.D.; previously involved in research on the efficacy of recreational soccer in men with prostate cancer undergoing androgen deprivation therapy and a member of the FCPC Steering Committee
	Dr. Svend Aage Madsen (SM)	Extensive knowledge and clinical experience in male psychology and gender-sensitive health promotion	Personal email	Telephone interview	Licensed specialist in psychotherapy, Ph.D.; co-founder and board member of the Danish Network on Research in Men and Masculinities and co-founder and president of the Danish Men’s Health Society
	Professor Michael Borre (MB)	Extensive experience with clinical research and medical treatment of prostate cancer patients	Personal email	Office, Health Research Department, Copenhagen University Hospital, Rigshospitalet	Professor, senior consultant/resident, D.M.Sc., M.D., Ph.D.; chair of the executive board of the Danish Multidisciplinary Cancer Group (DMCG) and the Danish Prostate Cancer Group (DaProCa)

**Table 2:** Sampling, recruitment, and characteristics of stakeholders in step 2

	Informants	Sampling criteria	Recruitment	Interview setting	Informant characteristics
STEP 2 STAKEHOLDERS (*n* = 17) (focus group interviews)	Urology nurses	Authority on and responsibility for referral of men with prostate cancer to rehabilitation, including assessment of rehabilitation needs	Personal email to the leader of the nursing team at the Copenhagen Prostate Cancer Center, Copenhagen University Hospital Rigshospitalet	Nurses’ office, Department of Urology, Copenhagen University Hospital Rigshospitalet	Five (*n* = 5) female nurses, including the team leader; participants had from 7 months to 12 years’ experience working with men with prostate cancer
	Soccer coaches	Experience with non-professional soccer coaching and/or management of a local soccer club	Email invitation distributed via the Danish Football Association to secretaries and/or non-professional coaches of local soccer clubs in the Copenhagen area	Common room, local soccer club in the Copenhagen area	Five (*n =* 5) volunteers (31–69 years of age) from two different soccer clubs, including one female club president and four non-professional male coaches
	Prostate cancer patients	Personal experience with prostate cancer regardless of treatment status, disease status and previous soccer experience	Verbal invitation to patients participating in a hospital-based patient education at Frederiksberg Hospital and written invitation distributed via email to members of the Danish Prostate Cancer Patient Association	Office, Health Research Department, Copenhagen University Hospital Rigshospitalet	Seven (*n =* 7) men with prostate cancer (time since diagnosis: 4 months to 5 years); five of the men (56–71 years of age) had played soccer previously while two had no prior soccer experience

### Data Collection

#### Key Informant Interviews with Experts (Step 1)

ML, who is a trained health professional (certified occupational therapist), served as interviewer and used a semi-structured format that included a standard open-ended question: “According to you, as an expert, what should be included in a curriculum for training non-professional soccer coaches in the delivery of community-based soccer in men with prostate cancer?”. This question was followed by a number of questions on specific suggestions thematically related to the individual key informant’s area of expertise (e.g., “How should the soccer training be structured?”, “How can the coach create the ideal psychosocial environment for male patients?”, and “How should the coach manage physical complaints from the participants?”). The interviews lasted approximately 45 min and were audio recorded. Within 1 week after the interview, informants received interview transcripts and a condensed summary for validation (member checking) including the opportunity to provide supplementary comments.

#### Focus Group Interviews with Stakeholders (Step 2)

The focus groups interviews were facilitated by ML, while NMH and JM assisted ML as observers and took notes during the interviews. The focus group participants were presented with the outline of the curriculum developed on the basis of the inputs from the key informants. The presentation included this open-ended question: “How confident are you that the preliminary content of this curriculum will assist non-clinical soccer coaches in the delivery/provision of a motivating, safe and effective soccer intervention for men with prostate cancer?”, followed by a standard set of questions for each focus group with some modifications related to the specific role of the stakeholders, such as referral of patients (by clinical nurse specialists), management of the intervention (by non-professional soccer coaches and club representatives), and sustainment of potential participants (men with prostate cancer). Lasting approximately 90 min each, the interviews were audio recorded and transcribed my ML. The coaches and club representatives were given a small gift valued at USD 20.

### Data Analysis

The key informant and the focus group interviews were analyzed separately using a thematic analysis, as described by Braun and Clarke [17]. First, the verbal data (recorded interviews) were transcribed into Microsoft Word documents, which were read repeatedly and coded informally (notes were taken and ideas for coding were marked down) by two researchers (ML and JM) independently. Second, ML systematically identified features in the data related to the purpose of the interviews by organizing the text into meaningful groups (codes) using NVivo 9.0 (QSR International). After all the data had been coded and collated, we developed broader themes by analyzing each code, including how they could be combined to form a theme. In the next step, we defined each sub-theme and overarching themes by identifying its essential characteristics and by determining what aspect of the data each theme captured. Lastly, we wrote up the analysis. For the focus group interviews, we also chose particularly representative data extracts to serve as evidence of the themes.

### Ethics

All participants provided informed written consent prior to participation in the study. Key informants were informed that, given that they were professional experts, their contributions would not be anonymized, whereas stakeholders were assured all avenues had been taken to ensure anonymity and confidentiality. The FCPC trial has been approved by the National Committee on Health Research Ethics (file number H-2-2014-099). However, due to the nature of this particular sub-study and the low risk posed to participants, formal ethics committee approval was not required. The study was conducted in accordance with the Declaration of Helsinki.

## Results

### Step 1: Experts’ Perspectives

Four themes emerged from the analysis of the key informant interviews: The Coach’s Qualifications, Structure of the Training, Prevention of Injuries, and A Non-Patient Environment, which informed development of the training course and intervention manual. Table 3 provides an overview of the findings from step 1.

**Table 3:** Overview of findings in step 1

Sub-themes	Overarching themes	Summary of proposed learning goals
Empathy and potential identification	Theme 1 The Coach’s Qualifications	- Learning about the disease (prostate cancer and treatment program) - Personal role of the coach (being an empathetic authority) - Knowledge of how to support the health-promoting effects of the soccer training - Theory and practice for warming up to prevent injury (FIFA 11+) - Rules on the risk of injury and guidelines on participation by impaired players - Theory on men, health- and disease-related behavior, including the importance of securing the men’s commitment, possibly through use of mentors
Authority		
Knowledge of prostate cancer and treatment		
Warm-up, exercises and games	Theme 2 Structure of the Training	
Warm-up (FIFA 11+)		
Autonomy and self-determination		
Risk groups (pain)	Theme 3 Prevention of Injuries	
Focus on training rather than matches		
Gentle start/instruction		
Establishing a commitment	Theme 4 A Non-Patient Environment	
Less focus on disease		

#### The Coach’s Qualifications

According to the experts, the coach must be empathetic, able to motivate and keep the men on the program, and able to generate team spirit. Moreover, the coaches should be able to differentiate the training so that the weakest members of the group, who may also be the ones in greatest need of the intervention, do not fall by the wayside. The two experienced prostate cancer physicians (Prof. Klaus Brasso (KB) and Prof. Michael Borre (MB)) emphasized that the coaches should have a basic knowledge of prostate cancer, including knowledge of side effects such as weight gain, weak bones, fatigue, and incontinence, and understand the significance of these for the soccer training.

#### Structure of the Training

Prof. of Team Sport and Health Peter Krustrup (PK) recommended that the training should be divided into three parts: warm-up, exercises, and games. Specifically, PK suggested that the warm-up session should include coordination exercises, balance exercises, strength exercises, and easy ball exercises. Warm-up should be followed by a practice session that can be adapted to the level of the players and that some exercises should be left out, such as using the soles of the feet to maneuver the ball and headers. The last part of the training could be rounded off with a game. There should be scope for everyone to try and shoot a goal, which could be ensured by arranging small-scale three-a-side or five-a-side games. For matches, PK suggested using soft, lightweight balls that are easier to play with. The optimal training session length, according to PK, is around 60 min. Dr. Svend Aage Madsen (SM), specialist in psychotherapy and men’s health, recommended that the coach should be in charge of how the training is structured. However, at the same time, SM stressed that the men should be autonomous and have a say in decisions.

#### Prevention of Injuries

All experts underlined the need to take things slowly in the initial phase of the intervention to avoid injury and muscle pain that might be mistaken for symptoms of the disease, which would lead to unnecessary visits to the hospital due to worry that the cancer has progressed. In the opinions of the clinical experts, it is important for the coaches to pay attention to each individual player’s physical condition on the day, including increased tiredness, increased fragility, and focus on the disease, which could be a consequence of a change in treatment regime or a worsening of the disease. Furthermore, both clinical experts pointed out that patients undergoing palliative radiotherapy constitute a particularly high-risk group, however also unlikely to turn up for soccer training expecting a certain natural selection to occur.

PK stated that the focus should be on training rather than playing matches, since it is safer, and that it would be appropriate to look at FIFA 11+, an injury-prevention warm-up program, for inspiration. Also, PK suggested it would be a good idea to introduce rules about the players not stepping on the ball to tame it or while dribbling. PK also suggested drawing up guidelines as to when injured players may participate in training fully.

#### A Non-Patient Environment

According to SM, only a minority of men concern themselves with health. So it is important for a preventive measure such as soccer training to be a goal in itself—a shared social experience enjoyed in the company of others. MB pointed out that it might be a good idea, with a view to keeping the men on the program, to respect the men’s wish not to be burdened by talk of their illness and to use soccer to create a non-patient environment. SM further stressed that another important factor for retaining the men in the program is making them feel comfortable and welcome including setting up a commitment for the men through the soccer training, e.g., monitoring attendance and keeping an eye on individuals. SM suggested the use of mentors on the soccer team, with those who are skilled taking those who are less skilled under their wing, as this would create a mutual commitment.

### Step 2: Stakeholders’ Perspectives

The focus groups agreed with the key informants’ input and added the importance of clarifying the Responsibility of the Coach (Theme 1), the value of Positive Competition (Theme 2), and Social Inclusion (Theme 3) of the men in the club. Table 4 provides an overview of the findings in step 2.

**Table 4:** Overview of findings in step 2

Sub-themes	Overarching themes	Summary of adjustments of learning goals
Uncertain role	Theme 1 Responsibility of the Coach	- Clarity about safety hotline to professional experts in case of doubt - Motivation through sport (games, competition, interaction) - Personal feedback from previous players (men with prostate cancer) - Attention to and strategy for anti-stigmatization/inclusion in the club - Strategy (social activities) for keeping players with impairments in the program
Restitution		
Joining the team despite impairment		
Self-monitoring	Theme 2 Positive Competition	
Seriousness		
Humor		
Club within the club	Theme 3 Social Inclusion	
Being together on an equal footing and with shared understanding		

#### Responsibility of the Coach

According to the nurses, the coaches should be able to identify who needs extra attention and to put themselves in the shoes of a prostate cancer patient and should have the authority to tell the men to avoid maneuvers such as tackling, but otherwise leave the rest up to the men. The club representatives and the volunteer coaches were concerned with the question of responsibility and what role they should have. They stressed the need for clear instructions about whom to contact when in doubt:

I2: In some ways, if you’re entrusting the coaches with this kind of group, you’re giving them a lot of responsibility actually, since these are vulnerable people. So it’s important to have some kind of support hotline or whatever you might call it.

Furthermore, the soccer coaches described feeling that it is their responsibility to ensure that players who are suffering pain or injury stay with the team, and thus should get some guidance on taking the initiative to organize social activities (e.g., a team dinner), in which injured players can also take part.

#### Positive Competition

According to the men, it is important that the atmosphere between the players during training is fun. They should be allowed to joke and pull each other’s leg and to behave in the way men tend to do when they get together:

Ix: I take it the main thing is the social side. I mean, we’re not starting up a Premier League club. I want to join in, even though I’ve never played soccer because I realize that the important thing is to move around and work up a sweat. That’s what it’s basically about. It’s not about winning matches, is it? It’s about getting outside in the fresh air and romping around.

At the same time, the men stated that the training should have a certain level of seriousness:

I1: I completely agree that there should be certain expectations about the level. Because if it was just something we did just for fun and relaxation, it wouldn’t serve its purpose.

I8: There should be a bit of pressure – even though it shouldn’t be too obvious to those taking part – they should know that they’re expected to put in some effort (…).

I7: Maybe a bit of healthy competition.

In general the men with prostate cancer did not see injuries as a problem and stated that the coaches should not be overly cautious:

Ix: I don’t want to be treated like a patient; I’d rather be seen as a 71-year-old man and nothing else.

Ix: Yes, it’s easy for us to start feeling sorry for ourselves, so nobody else should start on that.

The men also expressed a desire to monitor their personal development by means of, for example, physical tests, also to keep up their motivation. Accordingly, according to the men, the coaches should be aware of providing feedback and setting personal targets for the individual players.

#### Social Inclusion

The club representatives and the volunteer coaches discussed whether playing in a team specifically for prostate cancer patients might stigmatize the players and they wonder whether it would be better to integrate the players into existing teams in the club:

Ix: The bad thing is that if you put together a team that consists of prostate cancer patients only, you’re kind of focusing on their illness. I think we should be careful not to do this; it’s certainly important. So it’s not like, oh here comes the prostate team, let’s go and watch them.

The men interviewed did however not see the risk of being stigmatized as being a problem:

I8: We’re getting to an age where we couldn’t care less what others think about us.

I9: I agree.

I6: Definitely.

Both the coaches and the men suggested inviting previous participants along to the training for the new coaches. They could give talks and share their specific experiences, providing the opportunity to ask someone who has been in the same situation questions.

#### Synthesis

Analysis of the results (including curriculum learning goals) from the interviews with experts and stakeholders resulted in the development of a training course and a written intervention manual. Hence, all coaches in charge of the FCPC intervention are required to fulfill a full 1-day training course (presented in Table 5). Coach candidates will receive course material 2 weeks ahead of their participation in the course. In addition to participation in the course, candidate coaches will be required to pass a first-aid course and will be expected to have prior extensive experience as either a player or coach.

**Table 5:** Final template and structure of the 1-day FCPC training course

Subject (classroom lecture or outdoor activity)	Responsible (presenter/instructor)	Duration (min)
Presentation of background to and history behind the FC Prostate Community trial	Project investigator	30
Knowledge of cancer in general and prostate cancer, including specific treatment of prostate cancer, side effects, and contraindications for physical activity	Medical doctor specializing in urology	90
Knowledge of what prostate cancer means in terms of everyday life	Clinical nurse specialist (urology)	30
Best practice session with soccer exercises and games	Soccer coach with experience in coaching men with prostate cancer	60
Practice session, injury-prevention warm-up concept FIFA 11+	Physiotherapist with soccer expertise	45
Theory and introduction of practice in field test (beep test and squat test)	Physiotherapist with soccer expertise	60
Instruction on procedures in connection with injuries	Project investigator	30
Personal feedback from soccer players with prostate cancer	Men with prostate cancer with experience from the FC Prostate intervention	30
Evaluation, questions, and rounding off the day	Project investigator	30

The written intervention manual clearly explains how the local coaches should structure and manage a training session: (Part 1) 20 min of warm-up exercises in accordance with the FIFA 11+ concept modified to suit older players [18]; (Part 2) 20 min of soccer exercises, including dribbling, passing, and shooting; and (Part 3) 20 min of 5–7-a-side soccer games. The coaches are encouraged to adhere to the basic structure, but are allowed to make changes to suit their group of men playing. Local coaches will use a web-based app to prospectively record fidelity to the intervention.

## Discussion

While it is widely acknowledged that the voluntary sector is a necessary and untapped resource in survivorship care [19, 20], literature on the optimal content and structure of the education of non-professionals to meet the exercise needs of cancer survivors is remarkably sparse. According to Bourke et al. [21], generalization of behaviors learned in supervised exercise environments to other contexts not supervised by professionals is pivotal in promoting habitual exercise in people living with and beyond cancer. By providing insights into the development and structure of a curriculum supporting safe delivery of a community-based exercise intervention in men with prostate cancer, we attempt to fulfill a previously identified gap in service for cancer survivors related to staff competency [22]. Hence, by ensuring that lay coaches recruited from local soccer clubs, gain special understanding and skills, we hope to contribute to increased availability of safe, low-cost, and easy accessible evidence-based exercise opportunities in the community as this may represent a more feasible approach to encouraging continued exercise in cancer rehabilitation.

One important issue, however, is how the curriculum can help the coach to manage the balance between, on the one hand, promoting a non-patient environment characterized by a lack of focus on the disease and, on the other, securing an intervention that is safe and respects the physical limitations and boundaries of the individual participant. As emphasized in Reed et al.’s [23] recent evaluation of a model for safe community exercise programming for people with chronic heart disease, the World Health Organization’s Global Action Plan on the Prevention and Control of Non-Communicable Diseases 2013–2020, specifically recommend “increased availability of safe environments in […] recreational spaces to encourage physical activity” (p. 51) [24]. However, as described by Clark et al. [25] in their study of cardiac rehabilitation graduates, patients living with a chronic disease may perceive instructors in the community as having limited knowledge and skills in relation to supervision of exercise in people with their condition. While patients with prostate cancer interviewed in our study stated that they specifically wish to avoid care and concern in the traditional sense, developing educational programs supporting safe, accessible exercise environments for patients with chronic diseases seem warranted. In this sense, and based on the findings in this study, the coach can be defined as a *lay professional* or *professional volunteer*, i.e., the coach is expected and requested to function as an authority and provide for the men’s safety while simultaneously serving as a soccer coach in the traditional sense and capitalizing on the setting outside the clinic/away from the hospital. This is important especially in the light of research describing that men opt out of rehabilitation because of a fear of losing control, as well as the fear that rehabilitation would invoke sympathy, cause dependency, and force them to confront death [26].

Another important issue is whether having special FCPC Prostate teams exclusively for men with prostate cancer carries the risk of leading to stigmatization. Theoretically, the fact that the men are brought together in a natural setting outside the hospital setting could in itself have an anti-stigmatizing effect in the sense that it involves integration into normal social culture. However, answering the question of how a special soccer team for cancer patients can be integrated into normal sports and club culture lies outside the realm of this study, and warrant further ethnographic inquiry. Specifically, informal interviews with club members, direct observation of interactions between club members (with and without prostate cancer), and analyses of formal and informal (electronic) documents (e.g., blogs posts, emails, minutes of meetings) could contribute to an understanding of barriers and facilitators in the integration of clinical subpopulations in traditional sports organizations. Another interesting finding of this study was the key experts’ and stakeholders’ identification of empathy and ability to generate team spirit as important instructor/coach qualifications. This is in line with the work by Heston and colleagues in relation to the development of a community-based physical activity program for survivors in the USA (i.e., LIVESTRONG at the YMCA) [22], stating that instructors are selected for having strong relationship-building skills including empathy and ability to develop relationships with and among survivors. Future studies are needed to explore the self-efficacy of lay instructors and coaches including how they perceive the curriculum/educational program and adopt the intervention manual.

This study was conducted according to an approved qualitative research design, and the data and the analysis captured the intended focus of the study.

The study comprised a total of seven interviews with a total of 21 purposefully selected informants including four experts and three groups of different stakeholders. In both steps 1 and 2 of the study, themes identified by one (group of) informant(s) were introduced into subsequent interviews to enable theoretical sampling. To enhance credibility, we furthermore used initially identified descriptive codes in step 1 (i.e., expert interviews) as points for discussion in the focus group interviews with stakeholders in step 2. This not only allowed for a credibility check of the initial analysis but also allowed for continuous evaluation of achievement of adequate information power (i.e., saturation) [27]. Based on the degree of highly focused and high-density dialog between the interviewer and interviewees (step 1) and among the study participants (step 2), in addition to the high specificity of the study aim, we believe that the study has sufficient information power.

As with other qualitative studies, the findings were influenced by the researchers’ preconceptions including the intent to develop a curriculum for non-professionals based on the assumption that education of non-professionals is warranted, safe, and viable in sustainment of exercise behavior in clinical populations. However, with the aim of emphasizing reflexivity and trustworthiness, reflections and discussions about norms, practices, and values were maintained throughout the overall process.

## Conclusions

This study does not attempt to provide a general set of rules or safety measures to promote or optimize the delivery of community-based exercise in cancer survivors, and the specific inputs provided by the experts and stakeholders may only be of relevance to the delivery of community-based soccer in men with prostate cancer. However, the general principles related to the necessary clarification of the coach’s responsibility in relation to the prevention and management of injuries and participant adherence through a non-patient environment may be transferable to the training and education of other groups of lay persons in charge of delivering exercise interventions to other clinical subpopulations in a non-hospital setting.

## Abbreviation

FCPC: FC Prostate Community
